# Predictors of Survival in Elderly Patients with Metastatic Colon Cancer: A Population-Based Cohort Study

**DOI:** 10.3390/cancers14215208

**Published:** 2022-10-24

**Authors:** Bogdan Badic, Anne-Marie Bouvier, Véronique Bouvier, Marie Morvan, Valérie Jooste, Arnaud Alves, Jean-Baptiste Nousbaum, Noémi Reboux

**Affiliations:** 1 Digestive Surgery Department, CHRU Brest, 29200 Brest, France; 2 Registre des Cancers Digestifs du Finistère, 29609 Brest, France; 3 LaTIM-Laboratory of Medical Information Processing, INSERM UMR 1101, Université Bretagne Occidentale, 29238 Brest, France; 4 Digestive Cancer Registry of Burgundy, Dijon University Hospital, INSERM UMR 1231 EPICAD, Medical School, University of Burgundy-Franche Comté, 21000 Dijon, France; 5 Digestive Cancer Registry of Calvados, Caen University Hospital, ‘ANTICIPE’ U1086 INSERM-UCN, Normandie University UNICAEN, Centre François Baclesse, 14000 Caen, France; 6 EA7479 SPURBO, Université de Bretagne Occidentale, 29200 Brest, France; 7 CHRU Brest, Service d’Hépato-gastro-entérologie, 29200 Brest, France

**Keywords:** stage-4 colon cancer, elderly, surgery, chemotherapy, survival

## Abstract

**Simple Summary:**

In addition to the age and comorbidities of elderly patients, the presence of a metastatic disease makes the choice of therapeutic options difficult. Our retrospective study aimed to determine the predictive factors for survival in patients aged 80 years and older with metastatic colon cancer. We analyzed data of 1115 patients issued from four digestive tumor registries. Comorbidity burden, metastasis location, chemotherapy, and curative surgery of primary tumors and metastases are prognostic factors correlated with overall survival. Although this study is retrospective, our data were collected across multiple sites and reflect the outcomes of the management of elderly patients with metastatic colon cancer.

**Abstract:**

Oncological strategies in the elderly population are debated. The objective of this study was to determine the predictive factors of survival in patients aged 80 years and older with metastatic colon cancer. Data from four digestive tumour registry databases were used in this analysis. This population-based retrospective study included 1115 patients aged 80 years and older with stage IV colon adenocarcinoma diagnosed between 2007 and 2016. Cox regression was used to assess the impact of different prognostic factors. Age was significantly correlated with the surgical treatment (*p* < 0.001) but not with overall survival. Patients with a low comorbidity burden had better survival than patients with higher comorbidities scores (9.4 (0–123) versus 7.9 (0–115) months) (*p* = 0.03). Surgery was more common for proximal colon cancer (*p* < 0.001), but the location of the primary lesion was not correlated with improved survival (*p* = 0.07). Patients with lung metastases had a better prognosis than those with liver metastases (HR 0.56 95% CI 0.40, 0.77 *p* < 0.001); multiple organ involvement had the worst survival (HR 1.32 95% CI 1.15, 1.51 *p* < 0.001). Chemotherapy was associated with improved survival for both operated (HR 0.45 95% CI 0.35, 0.58 *p* < 0.001) and non-operated patients (HR 0.41 95% CI 0.34, 0.50 *p* < 0.001). The majority of patients receiving adjuvant treatment had a low comorbidity burden. In our study, the location of metastases but not the primary tumor location had an impact on overall survival. Low comorbidity burden, curative surgery, and chemotherapy had a significant advantage for elderly patients with metastatic colon cancer.

## 1. Introduction

Worldwide, with more than 1.9 million new cases of colorectal cancer (CRC) and an estimated 935,000 deaths in 2020, colorectal cancer is the second leading cause of cancer death in developed countries [1]. In Europe, 21% of the population is over 60 years of age, and this proportion is increasing by almost 1% each year, making it the fastest-growing segment of the population [2]. The incidence rate of CRC increases rapidly with age, with an incidence of 90.2 per 100,000 for those aged 60 to 64 years, rising to 258.8 per 100,000 for those aged 85 and older [3,4].

In clinical practice, various scores are used to predict outcomes in patients with multiple comorbidities. One of the scores, the Charlson Comorbidity score (CCS), was used to assess the risk of death related to comorbidities. The CCS comprises 19 weighted comorbidities (such as cardiovascular disease, chronic kidney disease, uncomplicated diabetes, liver disease, etc.) according to the original definition [5]. It has been extensively studied and modified since its development [6].

The treatment of cancer should be tailored to the patient’s physiological age, tolerance to treatment, and life expectancy [7]. The presence of comorbidities, poor performance status, and concerns about toxicity and treatment efficacy may explain the high recurrence rates and higher mortality in older patients [8]. Although adjuvant chemotherapy prolongs the time to recurrence, the HIGHCARE project showed that the administration of chemotherapy was inversely correlated with age: in patients aged 70 to 79 years the risk of receiving chemotherapy was five times higher than in patients over 80, and four times lower than in patients under 60 (*p* < 0.001) [9].There is also an inter-country variability, with the FOLFOX/CAPOX chemotherapy regimen most used in Spain (68% of the administered chemotherapies), Estonia (64%), Poland (47%) and Portugal (42%) and 5-FU in Germany (50%) [9].

Furthermore, the outcome of major surgery in elderly patients may be similar to that of surgery in younger patients, with careful selection and a modified surgical procedure if necessary [10]. Elderly patients represent a heterogeneous group with discrepancies between chronologic and biologic age because of differences in functional and cognitive status, which limits comparison between the different series [11]. The majority of published studies on elderly patients with CRC have included patients over 70 or 75 years of age, but less data is available for older patients. The aim of this study was to determine the predictive factors for survival in patients aged 80 years and older with metastatic colon cancer.

## 2. Materials and Methods

### 2.1. Data Collection

This study included patients 80 years and older, diagnosed with stage-IV colon adenocarcinoma from 1 January 2007 to 31 December 2016, which was the data cut off for the analysis. The data for this study were extracted from the four digestives cancer registry databases (Calvados, Côte d’Or, Finistère, and Saône-et-Loire), for tumour characteristics and treatments. For all cases were recorded, information on patient demographics, tumour characteristics, pathological staging, treatment modalities. Information was obtained from pathologists, general practitioners, hospitals, and private physicians (gastroenterologists, surgeons, and oncologists).

Registered parameters included age, gender, tumour location, clinical presentation, type of surgery, TNM staging (UICC 7th edition) [12], and chemotherapy. The Charlson comorbidity score (CCS) score was calculated for each patient taking into account all comorbid conditions [5]. As for tumour location, proximal colon tumours were defined as tumours localized in the caecum, ascending colon, hepatic flexure, or transverse colon. Distal colon tumours were defined as tumours situated in the splenic flexure, descending colon, or sigmoid colon.

### 2.2. Statistical Analysis

The analysis was performed using “R”, software environment version 4.1.1 (R Core Team). Qualitative data were described as number and frequency; quantitative data were described as median and range. Comparison of colon-cancer groups was performed by KHI2 or Fisher’s test for categorical data and Wilcoxon test for quantitative data. The Cochran Armitage exact trend test was used to test for trends in proportions. Overall survival (OS) was defined as the interval between the date of diagnosis and the date of death or last follow-up or time-point. Survival curves were established by the Kaplan–Meier method with comparison by the Log Rank test. A *p*-value < 0.05 was considered significant. A multivariate analysis on observed survival was performed, using Cox proportional hazards regression model.

### 2.3. Ethics Approval

The quality and completeness of each registry are certified every 4 years by an audit conducted by the National Institute of Health and Medical Research (INSERM), the National Public Health Institute (SpF), and the National Cancer Institute (INCa). The present study complied with our national and institutional guidelines and the ethical guidelines of the 1975 Declaration of Helsinki (6th revision, 2008) as reflected in a priori approval by the institution’s human research committee. This observational non-interventional study was approved by the French Data Protection Authority (CNIL, authorization n° 998024).

## 3. Results

### 3.1. Patient Characteristics

Table 1 presents the characteristics of all 1115 patients diagnosed with stage-IV colon adenocarcinoma between 2007 and 2016. The age ranged from 80 to 103 years, and 33 patients were 95 years or older. There were 620 (55.6%) women, and gender was not significantly related to the location of the primary tumour. Comorbidity burden was correlated with age (*p* < 0.002). For 733 (65.7%) patients, the diagnosis was disclosed by suggestive symptoms such as asthenia, weight loss, altered bowel transit, or anaemia. The most frequent tumour location was proximal (n = 611; 57%). Primary tumour location was not significantly correlated with the metastatic site (*p* = 0.79).

### 3.2. Treatment

Of the 629 (56.4%) operated patients, complete resection of primary tumour and metastases was realized for 23 (2%) patients, 251 (22.4%) patients had primary lesion but not metastases resection and 214 (19.1%) incomplete primary tumour and metastatic resection. Emergency surgery (in the context of tumour perforation or obstruction) was required for 275 (24.7%) patients. Surgery was more often realized in patients with emergency symptoms than in non-emergent patients (87.6 vs. 45.8%, *p* < 0.001), but at the expense of less extensive surgery (73.4 vs. 80.5%, *p* = 0.049). The rate of resection surgery decreased with increasing age (*p* < 0.001), 27.2% of 80- to 85-year-old patients received primary tumour resection, whereas the rate declined to 13.4% of patients age ≥ 90 years. Stenting procedures realized for 47 (4.2%) patients did not differ significantly between age groups (*p* = 0.73).

A total of 237 patients (21.4%) received chemotherapy, 150 (13.6%) patients were administered adjuvant chemotherapy, and 79 (7.1%) received chemotherapy alone. Regardless of the type of chemotherapy intended, rates of administration decreased with increasing age (*p* < 0.001). Among the 630 (56.4%) operated patients 137 (21.7%) patients received adjuvant chemotherapy, fluorouracil (FU)-based chemotherapy alone was used in *70* (11.1%) patients, 38 (6%) patients received doublet chemotherapy of FU *+* oxaliplatin, and 13 (2.0%) doublet chemotherapy of FU + irinotecan. The FU monotherapy was used in 31 (6.7%) non-operated patients, 26 (5.6%) patients received FU + oxaliplatin chemotherapy regimen, and 6 (1.3%) FU + irinotecan chemotherapy regimen. Targeted therapy was used in 21 (3.3%) operated and 9 (1.9%) non-operated patients.

Finally, best supportive care (BSC) was instituted for 535 (48%) patients, with a rate increasing with age (*p* < 0.001).

### 3.3. Survival and Prognostic Factors

By 31 December 2016, which was the data cut-off for this analysis, 98% patients had died. The median overall survival for the entire cohort was 9.1 months. Median follow-up was 4.2 months (range: 0–123.1).

**Figure 1:**
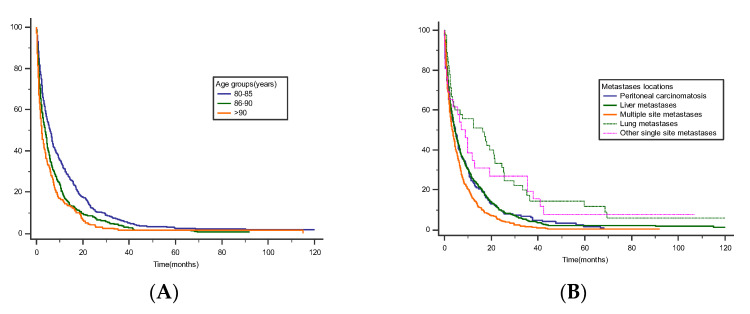
(**A**) Survival rates stratified by age groups (80–85 years (median 5.90, 95% CI 4.71 to 6.67), 86–90 years (median 3.71, 95% CI 3.00 to 4.45), >90 years (median 2.22, 95% CI 1.77 to 3.12), Log-rank *p* < 0.0001). (**B**) Survival rates stratified by metastases location (Peritoneal carcinomatosis (median 4.66, 95% CI 3.29 to 5.90), Liver metastases (median 4.54, 95% CI 3.83 to 5.36), Multiple site metastases (median 3.43, 95% CI 2.77 to 4.22), Lung metastases (median 16.29, 95% CI 3.90 to 21.35), Other single site metastases (median 8.96, 95% CI 2.35 to 12.93), Log-rank *p* < 0.0001).

The 1-year mortality rate after diagnosis was 79.5%. The mortality rate increased with patient age (70.5% in patients aged 80–85 years, 93.8% in patients aged 86–89 years, and 100% in patients above 90 years, (*p* < 0.001)). The 3-years and 5-years mortality rate were 95.6% (93.8% in patients aged 80–85 years, 96.6% in patients aged 86–89 years) and, respectively, 98.7% (98.2% in patients aged 80–85 years, 98.7% in patients aged 86–89 years) (Figure 1A).

Univariate analysis revealed improved OS for patients with low comorbidity burden (CCS ≤ 1) compared to those with higher comorbidities scores (*p* = 0.03) (mean OS of 9.4 (0–123) months versus 7.9 (0–115) months, respectively). Age was not significantly correlated with the comorbidity burden (*p* = 0.46).

Patients with lung metastases (HR 0.56 95% CI 0.40, 0.77 *p* < 0.001) had better prognosis than those with liver metastases. Conversely, patients with multi-organ metastases (HR 1.32 95% CI 0.15, 0.51 *p* < 0.001) had poorer survival (mean OS of 6.5 (0–43) months) than those with peritoneal metastases (mean OS of 9.4 (0–41) months) (Figure 1B) (Table 2).

**Figure 2:**
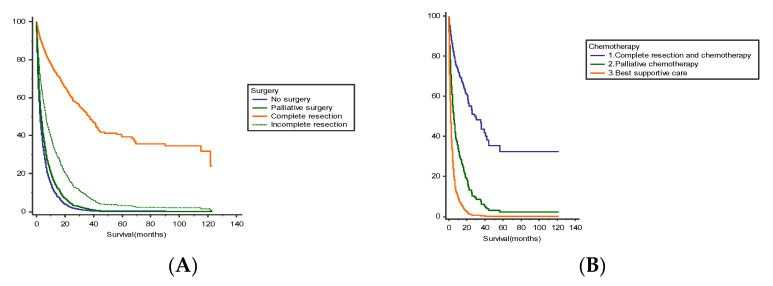
(**A**) Survival rates stratified by type of surgery (no surgery (median 2.45, 95% CI 2.16 to 2.80); palliative surgery (derivative stoma, internal bypass) (median 3.13, 95% CI 2.13 to 4.43); complete resection (complete resection of primary tumor and metastases) (median 36.40, 95% CI 18.16 to 123.06); Incomplete resection (incomplete resection of primary tumor and metastases) (median 7.32, 95% CI 6.38 to 8.80) Log-rank *p* < 0.0001). (**B**) Survival rates stratified by chemotherapy (complete resection of primary lesion and adjuvant chemotherapy (median 36.10, 95% CI 18.16 to 56.20); palliative chemotherapy (median 7.96, 95% CI 6.76 to 10.00); best supportive care (median 2.06, 95% CI 1.80 to 2.32) Log-rank *p* < 0.0001).

Surgery was more common for proximal colon cancer than for distal colon cancer (*p* < 0.001) but the location of the primary lesion was not correlated with better survival (*p* = 0.07). Survival was higher in operated patients: median OS of 5.8 (0–106) months for patients who underwent complete resection of primary lesion and metastases, 3.7 (0–115) months for palliative surgery (derivative stoma, internal bypass), and 1.4 (0–32) months for non-operated patients (*p* = 0.01). Postoperative mortality (30-days mortality) was 67.4% (Figure 2A). The analysis of overall survival of patients operated on for three periods of time (2007–2010; 2010–2013; 2013–2016) did not reveal significant differences (*p* = 0.45).

Chemotherapy was associated with better survival for both operated (HR 0.45 95% CI 0.35, 0.58 *p* < 0.001) and non-operated patients (HR 0.41 95% CI 0.34, 0.50 *p* < 0.001). Complete resection of primary tumour and metastases associated with adjuvant chemotherapy (HR 0.14 95% CI 0.07, 0.26 *p* < 0.001) has improved OS compared to adjuvant chemotherapy et primary tumour resection only (HR 0.29 95% CI 0.23, 0.38 *p* < 0.001 or incomplete resection of primary lesion and metastases (HR 0.31 95% CI 0.24, 0.72 *p* < 0.001) (Figure 2B). For patients with primary tumour resection and adjuvant chemotherapy, FOLFOX regimen and targeted therapy significantly improved OS (27.0 months; 95% CI 13.6, 34.9) compared to FU monotherapy (19.8 months; 95% CI 11.6, 22.1) or FOLFOX regimen (21.8 months; 95% CI 11.0, 23.3). The majority of patients receiving adjuvant treatment had a low comorbidity burden.

Multivariate analysis showed that comorbidity burden was associated with OS (Charlson score = 1 HR 0.77 95% CI 0.69 to 0.91 *p* = 0.001). The location of the metastases was also a prognostic factor, with lung metastases characterized by better survival (HR 0.46 95% CI 0.32 to 0.66 *p* < 0.001) than peritoneal carcinomatosis, whereas multi-organ metastases tended to decrease survival. Finally, chemotherapy (HR 0.52 95% CI 0.40 to 0.67 *p* < 0.001) and surgery (complete resection of primary tumour and metastases HR 0.30 95% CI 0.24 to 0.38 *p* < 0.001), and, especially, the combination of the both (HR 0.22 95% CI 0.12 to 0.42 *p* < 0.0001), had the strongest impact on the patient’s overall survival.

Multivariate analysis showed no significant effect of gender and age on overall survival.

## 4. Discussion

In our study, the comorbidity burden, curative surgery, chemotherapy, and the location of metastases had an impact on overall survival. Curative surgery and chemotherapy improved OS but less than half of the patients received it.

Recent studies have shown approximately that 70% to 75% of patients with metastatic colorectal cancer survive beyond one year, 30% to 35% beyond three years, and fewer than 20% beyond five years from diagnosis [13]. Age is often considered a risk factor for in-hospital morbidity and mortality after colorectal surgery; there is compelling evidence that it is not the actual chronological age of the patient that is a risk for surgery but rather the quality of aging, comorbidity, and the functional status that defines the frailty state [14]. In our study, age and gender were not correlated with survival.

Significant comorbidities are independent risk factors for in-hospital morbidity and mortality after colorectal surgery in elderly patients. Emergency surgery is associated with poorer survival due to a higher perioperative mortality rate, related to more advanced tumour stage and poor physical status of patients at presentation [15]. Although elective oncologic surgery offers the same life expectancy as for younger patients [16], older patients are more likely to have more advanced disease and to undergo emergency surgery, and less likely to undergo curative surgery than younger patients [15]. With comparable postoperative complication rates between younger and older patients (28.7% versus 32.3%) and a postoperative mortality rate below 5%, liver resection for colon metastases in patients aged over 70 years of age can offer a reasonable 3-year survival rate [17].

Consistent with the previous literature, we found that the location of the metastases is also important, with decreased survival when they are located in multiple organs, and increased survival when they are in the lungs compared with the peritoneal cavity or the liver [18,19]. An analysis of the South Australian Registry for advanced colorectal cancer showed similar results with an overall survival of 41.1 months for the lung-only metastases group, followed by the liver- and pelvic-only metastases groups (22.8 and 23.8 months, respectively) [20]. Differences in metastatic sites might be partly responsible for differences in survival between primary tumour locations. A nationwide autopsy study of 5817 patients showed a higher rate of liver metastases and lung metastases in patients with distal CCR, whereas proximal CCR was associated with a higher rate of peritoneal metastases and metastases at other sites [21].

Palliative surgery is indicated for most patients with bowel obstruction or uncontrollable bleeding, at the cost of high mortality and long hospitalization relative to the patient’s remaining survival time [22]. With an incidence between 15% to 29%, large bowel obstruction is associated with high postoperative morbidity and mortality and low 5-year survival [23]. Various studies have shown that complication rate, length of stay, readmission rates, and 1-year overall survival of patients undergoing urgent/emergent admission are higher compared to patients undergoing elective surgery [24].

In our study, adjuvant and palliative chemotherapy significantly improved overall survival. Although there is a higher rate of hematologic toxicity, standard colorectal cancer treatments offer similar benefits to younger patients [25]. In our series, about 21% of patients received chemotherapy. Similar to the results of other French cancer registries, our study showed an important increase in palliative chemotherapy in patients over 80 years old (18.91% versus 7.3%) over time [26]. These results are consistent with previous studies that showed that due to age and comorbidities, only a small and highly selected proportion of the elderly population received a full dose of treatment [27,28,29].

In our analysis, improved OS when fluorouracil (FU) was used in combination targeted therapy compared to FU monotherapy or associated with oxaliplatin. Reports of the effect of FU/oxaliplatin combination chemotherapy in patients over 70 years of age in clinical trials do not support its advantage over FU [30,31,32]. The FOCUS2 and FFCD 2001–02 trials compared fluoropyrimidine monotherapy with combination chemotherapy and showed an increase in the proportion of patients achieving an objective response and a small, nonsignificant increase in progression-free survival, but no difference in overall survival and higher incidence of grade 3–4 toxicity with combination chemotherapy [30]. In our series, only 21 (1.8%) patients received targeted therapy. The addition of bevacizumab to 5-fluorouracil monotherapy or doublet chemotherapy is well-tolerated and efficient in selected elderly patients with metastatic CRC [31]. Various studies have shown improved clinical outcomes in some elderly patients with anti-EGFR (epidermal growth factor receptor) therapies [27]. Geriatric assessment of cognitive function and autonomy impairment, predictive of severe toxicity or unexpected hospitalization, should be considered when making chemotherapy decisions [33].

Our study has several limitations. Despite the retrospective nature, our data were collected across multiple sites and described what is happening in the general population.

Another potential limitation is the long period of inclusion of patients, over 10 years, while the practices (chemotherapy, perioperative management, etc.) changed. Data regarding patient selection, results of surgery (postoperative complications, quality of life), or chemotherapy (tumour response, adverse effects) were not assessed because of the heterogenicity of populations. Finally, a widely used measure of comorbidity, the Charlson comorbidity score, was used in our study but other measurements of frailty can be used to reliably predict patient outcomes and response to potential therapies [28].

## 5. Conclusions

The comorbidity burden and location of metastases are associated with the overall survival of aged patients with metastatic colon cancer. The decision of treatment (surgery, chemotherapy, best supportive care) for these patients should take into account the health status and life expectancy of each patient. The risks and benefits of adjuvant therapy and surgery in elderly patients deserve further study.

## Figures and Tables

**Table 1:** Patient characteristics.

Characteristics	N = 1115
**Gender**	
Male	495 (44%)
Female	620 (56%)
**Age at diagnosis (years)**	85.0 (range 80.0, 103.1)
**Primary tumor location** (unknown 52 patients)	
Proximal Colon	611 (54.7%)
Distal Colon	452 (40.5%)
**Presenting features**	
Symptoms	733 (65.7%)
Emergency	275 (24.6%)
Fortuitous	97 (8.6%)
Unknown	10
**Charlson Comorbidity Score** (unknown 61 patients)	
0	509 (45.6%)
1	296 (26.5%)
2	143 (12.8%)
≥3	106 (9.5%)
**Metastatic sites** (unknown 1 patient)	
Liver	452 (40.5%)
Lungs	45 (4.0%)
Peritoneum	182 (16.3%)
Other (single location)	26 (2.3%)
Multiple locations	409 (36.6%)
**Treatment**	
Best supportive care	535 (48%)
Chemotherapy	237 (21.2%)
Palliative	79 (7%)
Postoperative (unknown 10 patients)	158 (14.1%)
**Surgery**	
Complete resection of primary tumor and metastases	23 (2%)
Complete resection of primary tumor	251 (22.4%)
Partial resection of primary tumor and metastases *	214 (19.1%)
Palliative surgery (surgical bypass, diverting ostomy)	112 (10%)
Exploratory laparotomy	29 (2.5%)

* Partial resection of primary tumor and metastases-incomplete resection (R1/R2) of primary tumour and incomplete resection of metastases.

**Table 2:** Survival and Prognostic Factors.

Characteristics	Univariate Analysis			Multivariate Analysis		
	HR ^1^	95% CI ^1^	*p*-Value	HR ^1^	95% CI ^1^	*p*-Value
**Gender**						
Male	—	—				
Female	1.10	0.98, 1.24	0.12			
**Age at diagnosis**						
80–85	—	—		—	—	
86–90	1.35	1.18, 1.54	<0.001	1.11	0.91, 1.35	0.3
≥90	1.65	1.40, 1.90	<0.001	1.03	0.82, 1.30	0.8
**Charlson comorbidity score**						
0	—	—		—	—	
1	0.84	0.73, 0.96	0.01	0.77	0.69, 0.91	0.001
2	1.04	0.86, 1.25	0.7	0.98	0.80, 1.21	0.9
≥3	1.19	0.96, 1.47	0.11	1.06	0.84, 1.34	0.6
**Emergency symptoms**						
No	—	—				
Yes	0.96	0.84, 1.11	0.6			
**Primary tumour location**						
Proximal	—	—				
Distal	0.89	0.79, 1.01	0.068			
**Metastasis sites**						
Liver	—	—		—	—	
Lungs	0.56	0.40, 0.77	<0.001	0.46	0.32, 0.66	<0.001
Peritoneum	1.04	0.87, 1.23	0.7	0.99	0.81, 1.21	>0.9
Other (single location)	0.64	0.42, 0.96	0.033	0.74	0.48, 1.14	0.2
Multiple locations	1.32	1.15, 1.51	<0.001	1.29	1.10, 1.51	0.001
**Treatment**						
Chemotherapy						
Best supportive care	—	—		—	—	
Adjuvant Chemotherapy	0.23	0.19, 0.28	<0.001	0.16	0.13, 0.21	<0.001
Palliative chemotherapy	0.45	0.35, 0.57	<0.001	0.39	0.31, 0.51	<0.001
**Surgery**						
Complete resection of primary tumor and metastases	0.24	0.14, 0.41	<0.001	0.30	0.24, 0.38	<0.001
Complete resection of primary tumor	0.82	0.68, 0.99	<0.001	0.46	0.39, 0.54	<0.001
Partial resection of primary tumor and metastases	0.56	0.48, 0.66	<0.001	0.62	0.53, 0.74	<0.001
Palliative surgery (surgical bypass, diverting ostomy)	0.75	0.52, 1.1	0.14	0.73	0.59, 0.90	0.04

^1^ HR = hazard ratio, CI = confidence interval.

## Data Availability

On request to corresponding author, upon reasonable request.
